# Encoding CO_2_ Adsorption in Sodium Zirconate by Neutron Diffraction

**DOI:** 10.3390/molecules29163798

**Published:** 2024-08-10

**Authors:** Connor Gammie, Fabian Hesse, Blair Kennedy, Jan-Willem G. Bos, Aimaro Sanna

**Affiliations:** 1 Institute of Mechanical, Process and Energy Engineering, School of Engineering and Physical Sciences, Heriot-Watt University, Edinburgh EH14 4AS, UK; 2 Institute of Chemical Sciences and Centre for Advanced Energy Storage and Recovery, School of Engineering and Physical Sciences, Heriot-Watt University, Edinburgh EH14 4AS, UK; fabian-hesse89@gmx.de; 3 School of Chemistry, St Andrews University, St Andrews, N Haugh, St Andrews KY16 9ST, UK; bfk1@st-andrews.ac.uk (B.K.); j.w.g.bos@st-andrews.ac.uk (J.-W.G.B.)

**Keywords:** carbon dioxide sorbent, sodium zirconate, neutron diffraction, TGA, XRD, synthesis method, CO_2_ uptake, crystal structure

## Abstract

Recent research into sodium zirconate as a high-temperature CO_2_ sorbent has been extensive, but detailed knowledge of the material’s crystal structure during synthesis and carbon dioxide uptake remains limited. This study employs neutron diffraction (ND), thermogravimetric analysis (TGA), and X-ray diffraction (XRD) to explore these aspects. An improved synthesis method, involving the pre-drying and ball milling of raw materials, produced pure samples with average crystal sizes of 37–48 nm in the monoclinic phase. However, using a slower heating rate (1 °C/min) decreased the purity. Despite this, the 1 °C/min rate resulted in the highest CO_2_ uptake capacity (4.32 mmol CO_2_/g Na_2_ZrO_3_) and CO_2_ sorption rate (0.0017 mmol CO_2_/g) after 5 min at 700 °C. This was attributed to a larger presence of microstructure defects that facilitate Na diffusion from the core to the shell of the particles. An ND analysis showed that the conversion of Na_2_ZrO_3_ was complete under the studied conditions and that CO_2_ concentration significantly impacts the rate of CO_2_ absorption. The TGA results indicated that the reaction rate during CO_2_ sorption remained steady until full conversion due to the absorptive nature of the chemisorption process. During the sorbent reforming step, ND revealed the disappearance of Na_2_O and ZrO_2_ as the zirconate phase reformed. However, trace amounts of Na_2_CO_3_ and ZrO_2_ remained after the cycles.

## 1. Introduction

Over the past 30 years, the effects of global warming and greenhouse gas production have become clear, leading to political action. In 2016, the Paris Agreement legally committed 186 of 197 UN parties to keep this century’s climate change below 2 °C, aiming for 1.5 °C. However, to meet this goal, the immediate large-scale deployment of Carbon Capture, Utilisation, and Storage (CCUS) is needed [1]. With regard to capturing technologies, there are three main solutions: Pre-Combustion capture (Pre-CC), Post-Combustion Capture (Post-CC) and Oxy-Combustion Capture (OCC) [2]. Most of the research is focused on low-temperature sorbents, which include amines, ionic liquids, and carbon materials [3,4,5,6,7]. Solid sorbents are becoming more favourable due to their ability to capture in a high temperature range, utilise waste heat for regeneration, and have the potential for being used in multiple cycles. CaO is the most used high-temperature CO_2_ sorbent due to its high capacity of 17.8 mmol CO_2_/g CaO, its low cost, and wide availability. However, its theoretical capacity is not achieved under real-world cycling conditions due to sintering and various treatments/modifications have been proposed to improve its performance [8]. Among the alternatives, sodium zirconate (Na_2_ZrO_3_) and lithium silicate (Li_4_SiO_4_) have shown good performance at high temperatures, with the former being the most appealing of the two due to the faster kinetics, better stability, and lower cost [9,10,11,12]. Until now, there has been a great deal of research into sodium zirconate as a sorbent [10,11,12]. Sodium zirconate can be produced via wet and solid-state reactions, with the latter method shown below [13]:

(1) $${Na}_{2}CO_{3}+ZrO_{2}\to \;{Na}_{2}{ZrO}_{3}+{CO}_{2}\;$$

With the subsequent carbonation reaction proceeding as follows:

(2) $${Na}_{2}{ZrO}_{3}+CO_{2}\to \;{Na}_{2}{CO}_{3}+{ZrO}_{2}\;$$

During the carbonation step, the uptake of CO_2_ (the theoretical uptake of 5.4 mmol CO_2_/g Na_2_ZrO_3_) occurs in two phases [14]. Phase one occurs at temperatures as low as 150 °C, at which sodium zirconate can adsorb at a low rate carbon dioxide until a sodium carbonate layer builds on the material surface. The layer eventually becomes too much of a hindrance to molecular interaction that adsorption stops. Phase two is the chemical absorption of CO_2_ following the reaction described by Equation (2). This occurs at temperatures greater than 550 °C, whereby the sodium ions required for reaction migrate through the shell that has developed to interact with the CO_2_. The rate of phase two is greater than phase one [15]. Mendoza-Nieto and co-workers (2022) proposed a novel CO_2_ sorption kinetic model on Na_2_ZrO_3_ through a consecutive reaction model, which considers a Na_2_CO_3_ layer that grows up by internal alkali ions diffusion instead of a parallel reaction proposed by the double exponential model [10]. A great deal of work has been conducted into the understanding of how the synthesis of the material affects the carbon dioxide uptake. Bamiduro et al. [13] produced sodium zirconate by means of spray drying, which formed smaller particles with minimised agglomeration and a more hollowed structure. This increased the surface area for interaction with carbon dioxide. The crystalline structure of the Na_2_ZrO_3_ obtained by the spray-dried method was the same as in the solid-state synthesis. Zhou et al. (2022) used a sol/gel and the freeze-drying route for the synthesis of Na_2_ZrO_3_ with a BET surface of 2.8 m^2^/g, which resulted in enhanced porosity and therefore, initial CO_2_ sorption, but also resulted in a reduced monoclinic phase, which limited the later CO_2_ uptake to 18.8 wt% indicating that the content of monoclinic crystal form was the most important determinant of sorption uptake capacity [16]. Munro et al. (2020) identified methods for the optimisation of the capturing performance of sodium zirconate by analysing various solid-state synthesis methods with ball milling achieving a BET surface of 5 m^2^/g, concluding that the synthesis conditions have a significant impact on CO_2_ uptake due to the tuning of the mineral phase purity and crystal size [17].

Despite the recent drive towards research into sodium zirconate, there is still a lack of knowledge on the detailed crystal structure of the material, both upon synthesis and upon carbon dioxide uptake and on the effect of phase purity in the sorbent performance. Many documents refer to a paper from 1994, where Bastow et al. describe monoclinic, orthorhombic, and hexagonal forms of sodium zirconate. A paper from 1968 reports the XRD pattern consistent with the hexagonal form but without reporting the structure [18]. They also mentioned how the monoclinic structure is assumed to be isostructural with lithium silicate_,_ although the peaks from an XRD test do not exactly match [19]. Cortes-Palacios et al. [20] synthesised sodium zirconate by solid-state synthesis starting from sodium acetate and Zirconium(IV) acetylacetonate, producing both monoclinic and hexagonal structures, although they did not mention much detail. Bamiduro [13] produced the monoclinic, hexagonal, and tetragonal forms of sodium zirconate. Ji et al. (2017) reported that monoclinic Na_2_ZrO_3_ was superior to hexagonal Na_2_ZrO_3_ in CO_2_ capture at high temperatures [21]. Pavan and Ling (2022) identified and quantified by synchrotron X-ray and neutron diffraction the solid-state phases involved in the carbonation/decarbonation cycle of Na_2_ZrO_3_ synthesised by solid-state synthesis in three stages (800 °C-12 h; 1000 °C-12 h; 1250 °C-4 h, with interstage grinding) [22]. The simultaneous presence of the monoclinic and tetragonal phases of ZrO_2_ previously identified was confirmed with the argument that the “metastable” tetragonal phase is favoured by smaller particles and can reincorporate into the Na_2_ZrO_3_ bulk, but the stable monoclinic phase does not, hindering the achievement of the theoretical maximum CO_2_ uptake capacity [22]. Recently, Chang et al. (2024) reported that the so-called hexagonal phase is actually monoclinic sodium zirconate with different types of disorder and that differences in the initial CO_2_ uptake are due mostly to the Na^+^ site occupancy differences and not to crystal structure differences [23].

A greater understanding of why different heating rates, and final synthesis temperature affect the resulting Na_2_ZrO_3_ purity and CO_2_ uptake performance is required to fully optimise the CO_2_ sorption using sodium zirconate. Building on previous research, this study is the first to investigate by in situ neutron diffraction the effects of the synthesis heating rate on the CO_2_ carbonation and desorption of sodium zirconate produced using an improved ball milling method. These samples were also analysed by standard XRD, TGA, and DTG to further the understanding of the CO_2_ sorption processes during carbon dioxide uptake.

## 2. Results and Discussion

### 2.1. X-ray Diffraction

The XRD analysis was conducted on the synthesised sorbents to verify their purity and establish the sodium zirconate mineral phases generated by the different synthesis methods and heating rates. The mineral phases were identified based on the literature [19,20].

**Figure 1:**
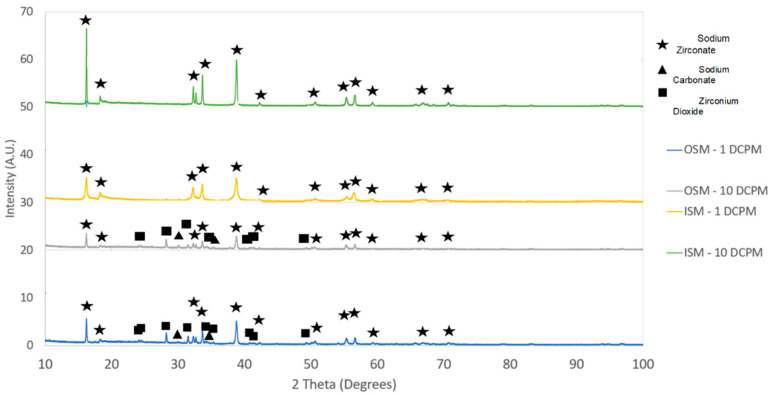
XRD results for original and improved synthesis methods.

**Figure 2:**
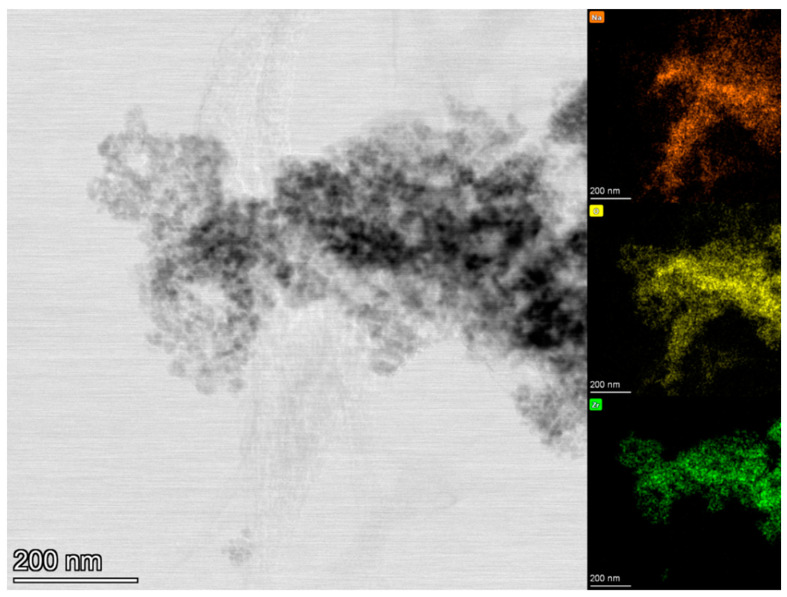
TEM of ISM—1 °C/min.

Figure 1 shows that monoclinic sodium zirconate is the only sodium zirconate phase for all the samples, with the improved synthesis method (ISM) samples clearly being purer compared to the old synthesis method (OSM) ones. The purity of the produced materials was estimated using the GSAS II software, where it can be seen that the sorbent produced by the ISM at 10 °C/min was the purer with a 100% monoclinic sodium zirconate phase (see Table 1). Contaminants such as sodium carbonate and zirconium dioxide were abundant in the OSM samples, while only sodium carbonate contamination was detected in the ISM obtained at 1 °C/min. This was confirmed by TEM as shown in Figure 2, where although there is a good correspondence of Na, O, and Zr, it can be appreciated the presence of some areas richer in sodium and oxygen. Therefore, the ISM samples were selected for the ND and TGA/DTG experiments.

The analysis of the diffraction patterns produced by the different heating rates shows differences in the structures between the heating rates. Looking at the OSM, the slower the heating rate, the more intense the peaks appear. It is understood that smaller crystals (smaller unit cell parameters) will produce wider peaks. The average crystal size of the sodium zirconates obtained by the ISM was obtained using the Scherrer equation. The crystal size increased by reducing the heating rate during the synthesis in the order: 10 °C/min (37.9 nm) < 1 °C/min (47.9 nm). The nanoparticle size was confirmed by TEM (see Figure 2), where the particles in the order of 20–40 nm are visible. The average grain size of the sodium zirconate decreases with increasing heating rate, which is in agreement with previous work, where also it is reported that the grain size is distributed in a broader range and microstructure orientation is significantly changed at the lower heating rate [24]. Therefore, the different heating rates affect the structure of the resultant sodium zirconates.

### 2.2. Neutron Diffraction Analysis

**Figure 3:**
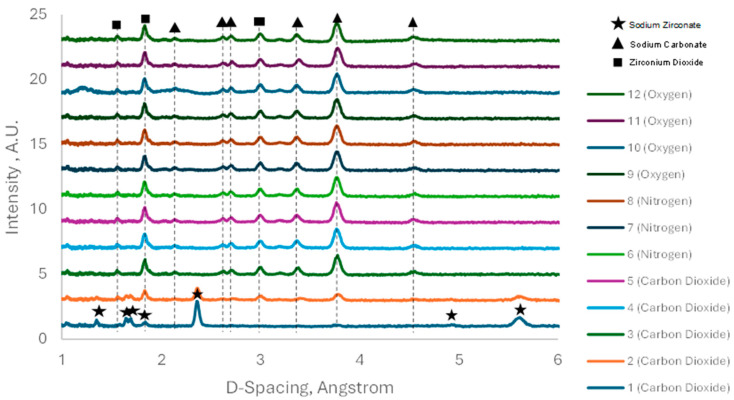
ISM—1 °C/min—neutron diffraction results from exposure to different gases. Readings taken each 5 min in presence of pure CO_2_.

In situ ND was then run to evaluate the change in the mineral phases under the reaction conditions. The first reading was taken immediately as the carbon dioxide gas was introduced and so represents the pure sample at 700 °C in N_2_. Figure 3 shows the ND patterns of the experiment run in the presence of the less pure Na_2_ZrO_3_ synthesised under the heating rate of 1 °C/min. Only monoclinic sodium zirconate was identified.

The sodium zirconate peaks that are present in reading 1 quickly disappear as the material reacts with the carbon dioxide, forming sodium carbonate (triangle) and zirconium dioxide (square). By the third reading, ten minutes after the first, it can be seen the zirconium dioxide peaks have completely disappeared. The sample was subjected to the carbon dioxide for another two readings; however, no further changes to the sample occurred—implying fast kinetics with full conversion between the five and ten minutes of exposure to the pure carbon dioxide gas. The material was then exposed to different gases to understand its behaviour at 700 °C. As can be seen in Figure 3, there appears to be no change at all during the exposure to nitrogen. The same is the case for the readings taken whilst exposed to oxygen. By the lack of changes to the material during the exposure to the different gases, it can be concluded that the material is stable at 700 °C, and does not release CO_2_ at this temperature under the conditions they were subjected to, although previous work shows that different carbonated Na_2_ZrO_3_ released CO_2_ at that temperature [17]. This can be ascribed to the higher temperature used here for the sorbent synthesis (900 °C).

**Figure 4:**
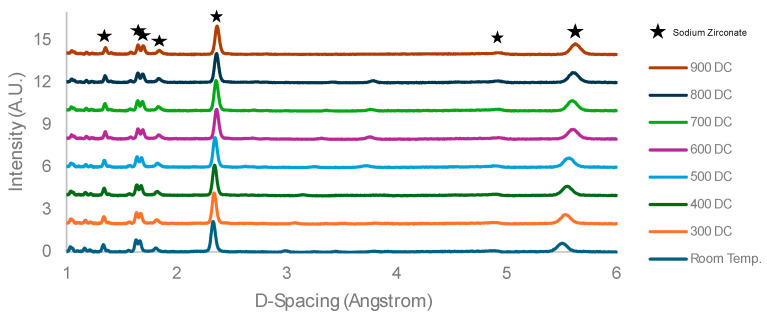
Sodium zirconate—10 °C/min—neutron diffraction results from heating from room temperature to 900 °C.

**Figure 5:**
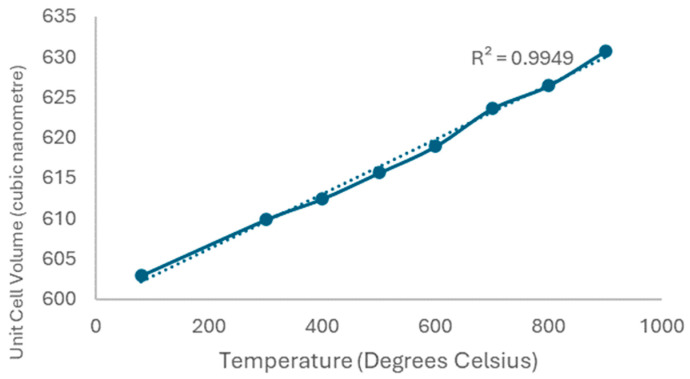
Unit cell volume change with temperature.

Figure 4 shows the ND curves of heating the purer 10 °C/min sodium zirconate from room temperature to 900 °C in N_2_. We can see from the shift in the peaks to the right that there is an increase in the crystal unit volume, as shown in Figure 5, where the solid increases in volume with temperature.

**Figure 6:**
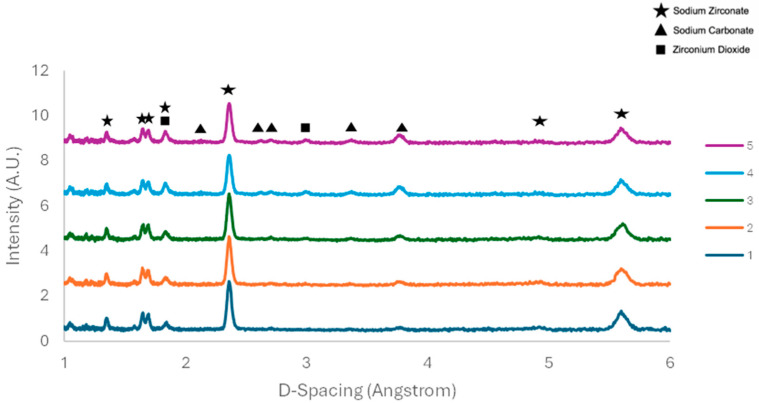
Sodium zirconate—10 °C/min—ND in presence of 10% CO_2_ (balance with N_2_). Readings each 20 min.

The sample produced by a 10 °C/min heating rate was then subjected to 10% carbon dioxide and 90% nitrogen. Readings were taken every five minutes; however, Figure 6 shows every fourth reading due to the slow nature of the reaction in the presence of a diluted CO_2_ stream. Figure 6 indicates the presence of key monoclinic sodium zirconate peaks at 1.4, 1.7, 2.3, and 5.6 Angstrom, which gradually reduced with carbonate peaks forming at 1.4, 1.8, 2.7, 3, 3.4, and 3.8 Angstrom.

**Figure 7:**
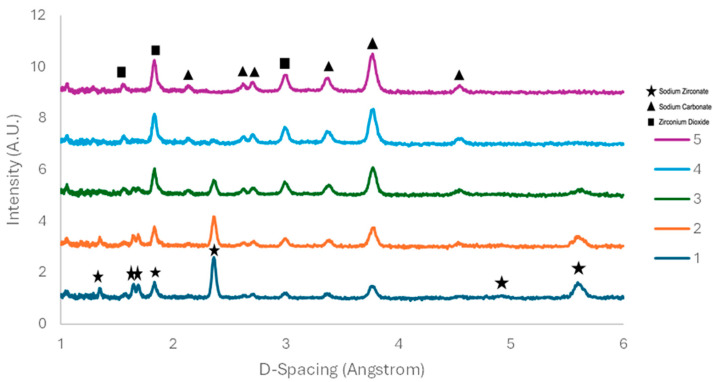
Sodium zirconate—10 °C/min—ND in presence of 50% CO_2_ (balance with N_2_). Readings taken each 5 min.

Due to the conversion rate of sodium zirconate being slow under 10% CO_2_ for the purpose of this study, the fraction of CO^2^ in the gas was increased to 50%. The results of this can be seen in Figure 7. Monoclinic sodium zirconate peaks present in reading 1 completely disappear in reading 6, indicating complete conversion to sodium carbonate/zirconium dioxide. The peak at 4.6 Angstrom also appears when the carbonation is almost complete.

**Figure 8:**
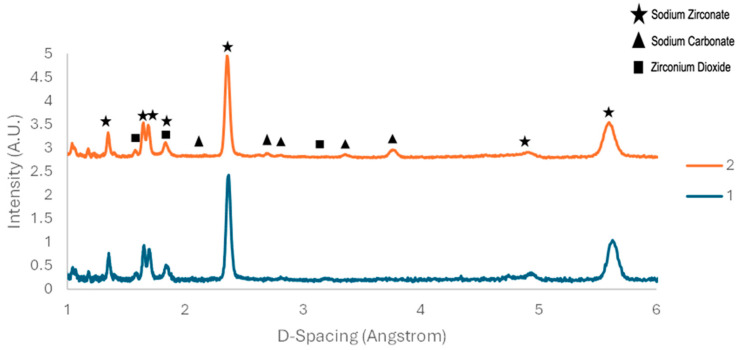
Comparison of sodium zirconate (1) before carbonation and (2) after reformation.

Previous work shows that the non-stochiometric excess of monoclinic ZrO_2_ persists after the regeneration stage [22]. This phenomenon was not detected in this work, since both monoclinic ZrO_2_ and Na_2_CO_3_ were present after the regeneration process as visible in Figure 3, Figure 7 and Figure 8.

Figure 8 compares the sodium zirconate before any carbonation occurs to the sodium zirconate that is reformed after the initial sample has been carbonated at 700 °C and reformed at 900 °C. It is clear that there is a great proportion of impurities present in the sample after it has reformed. The impurity peaks are mostly associated with sodium carbonate and zirconium dioxide due to the incomplete decomposition of the formed carbonate. The sodium carbonate peak may be due to how the material is reformed, and if there is sufficient contact area between the reactants as the material moves through its various phases (i.e., reduced contact due to agglomeration).

### 2.3. TGA-DTG Analysis

**Figure 9:**
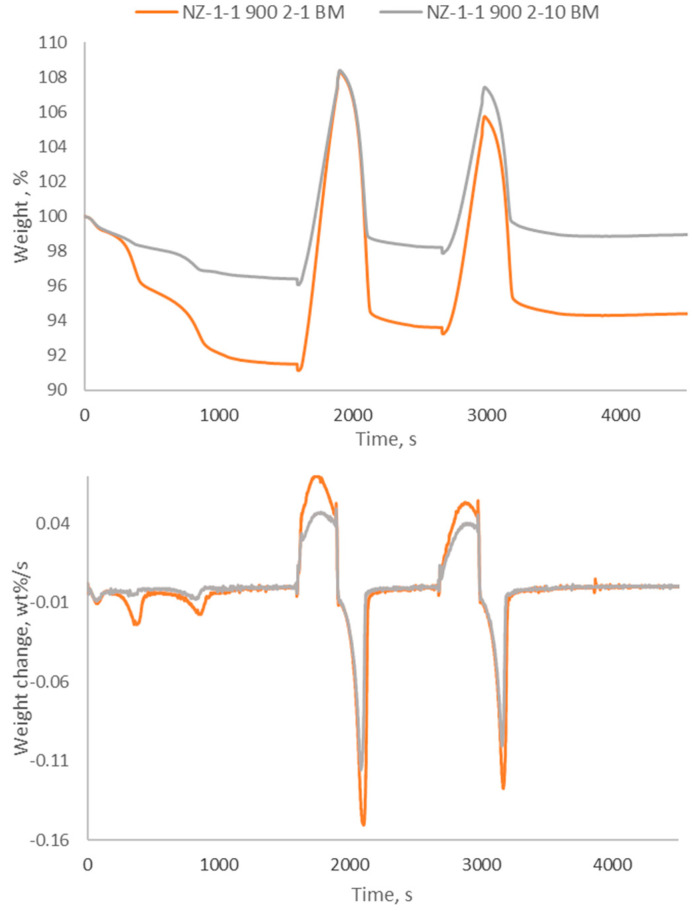
TGA and DTG Analysis of the ISM samples.

The sodium zirconates synthesised by the improved solid-state method were tested by TGA and DTG and the corresponding CO_2_ sorption curves for two consecutive cycles are shown in Figure 9. A short cycle of 5 min CO_2_ sorption was used for comparison reasons since the ND runs suggested that the bulk of the CO_2_ sorption occurs by fast absorption at the initial stage and only marginally improves later (see Figure 2) and this is also shown in the literature [17]. Various literary sources identify the initial adsorption phase, which occurs until the formation of the sodium carbonate layer prevents carbon dioxide gas from penetrating the material [15,17]. This is followed by a faster period of absorption, which occurs at higher temperatures, whereby the sodium ions migrate from the particle core through the carbonated shell for interaction with the CO_2_ gas. This phase can be ascribed to the first disappearance of Na_2_ZrO_3_ in readings 1–2 (Figure 3) and 1–3 (Figure 7). This is followed by a slow delayed crossing of the Na ions through the thicker carbonation product layer, which is not visible in the TGA tests due to the limited exposition time to CO_2_ (5 min). The data reported here are in agreement with the Mendoza-Nieto et al. (2022) CO_2_ sorption kinetic model where CO_2_ sorption occurs through the continuous formation of the Na_2_CO_3_ layer due to internal alkali ion diffusion [10].

The TGA tests were also used to evaluate the desorption stage that was not investigated by ND. It is clear that all the sodium zirconate sorbents follow a similar pattern whilst subjected to the conditions used here. Initially, the mass of the sorbents is reduced, with this stabilising as the temperature becomes constant at 900 °C. This mass reduces due to the removal of moisture and other adsorbed species in the presence of nitrogen. Upon the mass stabilising, the temperature is reduced to 700 °C and the gas composition is changed from pure nitrogen to 20% carbon dioxide (balanced by nitrogen). The mass increases drastically as the carbon dioxide reacts with the sodium zirconate to form sodium carbonate and zirconium dioxide, with the sorbent synthesised under a heating rate of 1 °C/min having faster sorption and larger CO_2_ uptake. The constant CO_2_ sorption rate excludes changes in the CO_2_ sorption mechanism, which may occur based on the previous literature due to extended exposure to carbon dioxide, which was not evaluated here [17].

During the initial heating, whilst the materials stabilise, the sample formed by a 1 °C/min heating rate loses the most weight when compared to the 10 °C/min sample, which does not change by more than 4%. The reason for this is that the slower heating rate results in a less pure sample with larger and irregular size distribution crystals (see the Scherrer analysis in XRD results), which allows more space in the material to diffuse and hold moisture. All the samples suffered from the reduction in mass moving through the cycles, not returning to their original values and becoming less effective with the second cycle. Table 2 shows the percentage change in weight for the two cycles and the two ISM sorbents. For comparison purposes, the sample synthesised by a 1 °C/min heating rate with the old method achieved a CO_2_ uptake of 14.2 wt% after 5 min absorption in the 1st cycle, which is 25% less than the uptake obtained using the improved method.

With regard to carbonation, more mass is gained when the synthesis heating rate is slower and the resulting NZ is less pure, which can be linked to the heterogeneous distribution of crystals obtained at the lowest heating rate and larger presence of micro defects that facilitates Na diffusion from the core to the shell of the particles. Having absorbed more carbon dioxide, the 1 °C/min sample also releases the most, however, it does return the furthest away from the original value (3.24% and 1.90%, respectively). The same trends are seen with the second cycle, with the slower heating rate resulting in a great carbon dioxide uptake, a greater desorption decrease, and the 1 °C/min sample also deviating furthest away from the mass before absorption. This behaviour is in line with previous observations, where a stabilisation of performance is obtained after 15–20 cycles [17].

The slower the heating rate, the greater the weight loss in the time period, and thus the 1 °C/min sample has the largest rate of change in weight. The 10 °C/min sample changes weight at a lower rate as visible in Figure 9. This is because of the different purity of the samples and their different microstructure. Furthermore, comparing the data in Figure 9 with those in Table 2, it appears that a minor content of sodium carbonate is beneficial to improved CO_2_ sorption performance, while a content of zirconium oxide seems to be detrimental. This could be linked to the fact that the sodium carbonate can facilitate the diffusion of the Na ions from the core of the particles to the surface at 700 °C and vice versa for the diffusion of the CO_2_ molecules. These are important observations to inform on high-temperature sorbent synthesis, which, based on these results, should contain a degree of carbonate contamination to enhance their CO_2_ absorption performance.

## 3. Materials and Methods

### 3.1. Materials

Zirconium dioxide in a purity of 99.9% and sodium carbonate in a purity of 99.6% were supplied by Sigma Aldrich.

#### 3.1.1. Original Synthesis Method (OSM)

The original synthesis method involved weighing out the calculated amount of reaction materials and grinding up in a mortar with a pestle for 15 min to ensure thorough mixing. The hand-mixed raw materials were then moved into a crucible and placed in a muffle furnace (Carbolite 1100, Carbolite Gero Ltd, Hope Valley, UK) for the required time. The purpose of the experiments was to vary the heating rate of the solid-state synthesis and determine the conditions that produced the purest sample. The synthesis conditions were as follows:

1. Method One—A heating rate of 1 °C /min up to a maximum temperature of 900 °C, then held at this temperature for two hours.
2. Method Two—A heating rate of 10 °C /min up to a maximum temperature of 900 °C, then held at this temperature for two hours.

After carrying out one set of results and analysing using XRD, it was established that the sodium zirconate was not as pure as required for further testing due to the mixing not fully combining the raw materials and so, a more effective mixing process was implemented.

#### 3.1.2. Improved Synthesis Method (ISM)

To improve the purity of the sodium zirconate, two aspects were considered: drying and mixing. One source of improvement was through the pre-drying of the raw materials. This was conducted in the ball-milled samples by heating the sodium carbonate in an oven at 200 °C for 12 h and the zirconium dioxide at 900 °C for 12 h. The reason for the lower temperature of sodium carbonate was that it can begin to decarbonate when exposed for a long time at temperatures above 850 °C following the reaction pathway below:

(3) $${Na}_{2}{CO}_{3}\to \;{Na}_{2}O+{CO}_{2}$$

**Figure 10:**
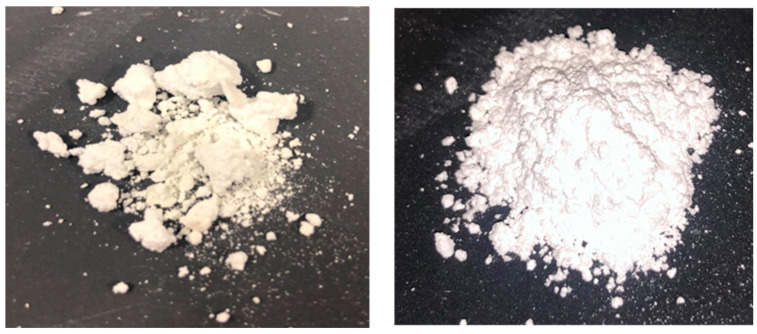
Synthesised sodium zirconate from original (**left**) [17] and improved (**right**) methods at 10 °C/min.

The drying removes the moisture, purifying the reactants further and removing potential side reactions. To improve mixing efficiency, a Fritsch Pulverisette planetary ball mill was used to mix the samples, alongside six sodium zirconate 12-millilitre grinding balls (10 mm in diameter) and four millilitres of isopropanol to prevent agglomeration during the milling process. A mixing time of five minutes followed by a rest time of five minutes for 12 cycles at 300 rotations per minute was used. The five minutes of rest time is required as a lot of heat is generated, and the breaks prevent overheating and premature reaction. The materials were then dried out in an oven for around two hours. The materials were then spread over two crucibles to allow for a thinner layer of material and achieve even heating, and finally calcined in the furnace using the same method reported in Section 3.1.1. This improved process meant a purer sample were achieved. Figure 10 show the sodium zirconate sample produced via the original and improved synthesis methods. It is clear from visual inspection that the powder is more homogeneous and less agglomerated than with the original synthesis procedure.

### 3.2. X-ray Diffraction

The X-ray diffraction (XRD) testing was conducted using a Nonius X8-Apex2 CCD diffractometer (Bruker, Billerica, MA, USA) with an Oxford Cryosystems Cryostream 1000 (which operates at around 100 K under normal operating conditions), an X-ray source with a copper anode (operating at 40 kV and 40 mA), and an energy-dispersive one-dimensional detector. XRD was used for establishing the purity of the samples and for the identification of phases in the sodium zirconate crystalline structures. The mean crystallites diameter size (nm) of the ball mill sodium zirconates was calculated using Scherrer’s formula (D = κλ/βCOSθ), where the Scherrer constant (k) for spherical crystallites with cubic symmetry is equal to 0.9, λ is wavelength (Cu = 1.5406 Å), β is the full width in radian at half maximum (FWHM) of the peaks, and θ is Bragg’s angle of the XRD peak.

### 3.3. Neutron Diffraction (ND)

Neutron diffraction testing was conducted at the ISIS Neutron Source using their General Materials diffractometer (GEM), UK [25]. The GEM software takes measurements over a period as requested by the user. It must also be noted that about 30 s is required between the measurements to allow the software to save the results. The sample rod contains two inserts—one is a thermocouple to measure the temperature and the other supplies the reaction gas. The sodium zirconate (5 g) powder was placed in the bottom of the rod and covered with a layer of mineral wool to stop the powder from being removed when subjected to pressure from the gas.

#### 3.3.1. Experiments Conducted on Sample Synthesised with 1 °C/min Heating Rate

The first experiment involved taking a room temperature reading of the sample under pure nitrogen conditions. This was then heated to 700 °C and another reading was taken. At 700 °C, the sample was subjected to pure carbon dioxide and readings were taken every five minutes. The sample was then subjected to pure nitrogen for several readings at five-minute intervals, and then under 20% oxygen (balanced by nitrogen) for several readings, again at five-minute intervals.

#### 3.3.2. Experiments Conducted on Sample Synthesised with 10 °C/min Heating Rate

The first reading on this sample was a room temperature study under pure nitrogen, as with the 1 °C/min sample. The sample was then heated up to 900 °C, with readings every 100 °C—it took 90 min to raise the temperature by this amount. This set of results was completed and finished with a reading at 700 °C as a cool-down reading. At 700 °C, the material was then exposed to a 10% carbon dioxide mixture, balanced by nitrogen, with readings every five minutes. Under these conditions, the conversion was too low and so was increased to 50% carbon dioxide_,_ again with readings every five minutes.

#### 3.3.3. ND Data Analysis

The raw data from the testing was analysed using the GSAS II version software package. The analysis was conducted by taking the raw data and refining it in such a way as to match the peaks of the neutron diffraction reading to the known pure samples from the Inorganic Crystal Structure Database (ICSD) [24]. Table 3 outlines the pure samples used and their key characteristics.

### 3.4. Thermogravimetric Analysis

The TGA equipment used in the experiment was a TGA/DSC 3+ Mettler Toledo. The exact conditions that the sodium zirconate sample was subjected to during the TGA testing were as follows:

1. Under the presence of pure nitrogen gas, heating until 900 °C and holding at this temperature initially for 27 min until the material stabilises.
2. Under 20 mol% CO_2_ (balanced by nitrogen), where the temperature is reduced to 700 °C and held for 5 min.
3. Under pure nitrogen, the temperature is again increased to 900 °C and held for 5 min.

This cycle was conducted and repeated once, giving two cycles of the conditions described for each sample. In the TGA experiment, gas was supplied at 60 cm^3^/min at a pressure of 1 bar. The experiments were run in triplicates with the error being <3%.

### 3.5. Derivative Thermogravimetry (DTG)

DTG was used to provide info on the rate of change in weight as time proceeds under the conditions and evaluate the reaction kinetics.

## 4. Conclusions

In this study, XRD, ND, TGA, and DTG were used to better understand sodium zirconate as a high-temperature carbon capture material. Improved solid-state synthesis was successfully conducted at two heating rates, 1 °C/min and 10 °C/min. The resulting material consisted of only the monoclinic phase (as confirmed by ND refinement), with relatively small traces of sodium carbonate and zirconium dioxide when synthesised at 1 °C/min. The crystals had a mean size between 37 and 48 nm. When the sodium zirconate sample synthesised at 1 °C/min was exposed to pure carbon dioxide at 700 °C, it fully converted to sodium carbonate and zirconium dioxide in under 10 min. However, with CO_2_ concentration reduced to 50%, full conversion required 15 min. Upon exposure to pure nitrogen and then pure oxygen at 700 °C, no further changes in composition or structure were detected. The sample synthesised at 10 °C/min underwent a variable temperature study, where the temperature was raised from room temperature to 900 °C. Throughout this temperature change, the structure of the sodium zirconate remained unchanged, but the unit cells increased in volume due to the added energy from the higher temperatures. This was identified by the peaks shifting to a higher D-spacing with the rising temperature, while the peak intensity remained constant. The XRD and ND results showed that a slower heating rate resulted in less pure sodium zirconate. This is due to the extended heating time allowing more interaction between the raw materials, leading to increased conversion and larger crystal sizes. The TGA showed that a slower heating rate during synthesis improved both the carbon dioxide uptake capacity and the uptake rate during the cyclic process. This improvement is primarily due to the larger structural defects in the lower-purity sodium zirconate formed at the lower heating rate. Overall, this study provides better insight into the crystalline structure of sodium zirconate during synthesis and its reaction with CO_2_. It also indicates that the tunability of sodium zirconate can be achieved through the improved synthesis method.

## Figures and Tables

**Table 1:** Comparison of synthesis methods (DCPM = degree Celsius per minute).

Sample	Na_2_ZrO_3_ wt%	ZrO_2_ wt%	Na_2_CO_3_ wt%
1 DCPM–OSM	66.9	21.4	11.6
10 DCPM–OSM	67.3	23.6	9.1
1 DCPM–ISM	84.7	0.8	14.5
10 DCPM–ISM	100	0	0

**Table 2:** CO_2_ uptake capacity and % changes from the TGA tests for the ISM sorbents.

Sample	CO_2_ Uptake Capacity after 1st Cycle, wt%	% Decrease after 1st Cycle	CO_2_ Uptake Capacity after 2nd Cycle, wt%	% Decrease after 2nd Cycle
ISM-1 °C/min	18.89%	13.92%	13.40%	10.83%
ISM-10 °C/min	12.77%	9.64%	9.69%	7.93%

**Table 3:** Summary of data from Inorganic Crystal Structure Database.

Sample	Space Group	Unit Cell Parameters				
		a	b	c	Beta	Volume
Sodium Zirconate	C 1 2/c 1	5.68623	9.85403	11.33008	99.903	625.391
Sodium Carbonate (low Temperature)	C 1 2/m 1	8.91799	5.2497	6.06909	100.949	278.693
Sodium Carbonate (High Temperature)	*p* 63/m m c	5.22204	6.71301	-	-	158.563
Zirconium Dioxide (Low Temperature)	*p* 1 21/c 1	5.20052	5.16255	5.32503	99.978	140.805
Zirconium Dioxide (High Temperature)	F m −3 m	5.11885	-	-	-	134.127

## Data Availability

Data will be made available upon request.

## References

[1] Martin-Roberts E., Scott V., Flude S., Johnson G., Haszeldine R.S., Gilfillan S. (2021). Carbon capture and storage at the end of a lost decade. One Earth.

[2] Hong W.Y. (2022). A techno-economic review on carbon capture, utilisation and storage systems for achieving a net-zero CO_2_ emissions future. Carbon Capture Sci. Technol..

[3] Wang Y., Memon M.Z., Seelro M.A., Fu W., Gao Y., Dong Y., Ji G. (2021). A review of CO_2_ sorbents for promoting hydrogen production in the sorption-enhanced steam reforming process. Int. J. Hydrogen Energy.

[4] Barrulas R.V., López-Iglesias C., Zanatta M., Casimiro T., Mármol G., Carrott M.R., García-González C.A., Corvo M.C. (2022). The AEROPILs Generation: Novel Poly(Ionic Liquid)-Based Aerogels for CO_2_ Capture. Int. J. Mol. Sci..

[5] Mohamed M.G., Samy M.M., Mansoure T.H., Li C.-J., Li W.-C., Chen J.-H., Zhang K., Kuo S.-W. (2022). Microporous Carbon and Carbon/Metal Composite Materials Derived from Bio-Benzoxazine-Linked Precursor for CO_2_ Capture and Energy Storage Applications. Int. J. Mol. Sci..

[6] Cheng J., Wu C., Gao W., Li H., Ma Y., Liu S., Yang D. (2022). CO_2_ Absorption Mechanism by the Deep Eutectic Solvents Formed by Monoethanolamine-Based Protic Ionic Liquid and Ethylene Glycol. Int. J. Mol. Sci..

[7] Luis P. (2016). Use of monoethanolamine (MEA) for CO_2_ capture in a global scenario: Consequences and alternatives. Desalination.

[8] Ji G., Yang H., Memon M.Z., Gao Y., Qu B., Fu W., Olguin G., Zhao M., Li A. (2020). Recent advances on kinetics of carbon dioxide capture using solid sorbents at elevated temperatures. Appl. Energy.

[9] Grasso M.L., Blanco M.V., Cova F., Gonzalez J.A., Arnedo Larochette P., Gennari F.C. (2018). Evaluation of the formation and carbon dioxide capture by Li_4_SiO_4_ using in situ synchtron powder X-ray diffraction studies. Phys. Chem. Chem. Phys..

[10] Mendoza-Nieto J.A., Martinez-Hernandez H., Pfeiffer H., Gomez-Garcia J.F. (2022). A new kinetic model for CO_2_ capture on sodium zirconate (Na_2_ZrO_3_): An analysis under different flow rates. J. CO2 Utiliz..

[11] Peltzer D., Hoyos L.A.S., Faroldi B., Munera J., Cornaglia L. (2020). Comparative study of lithium-based CO_2_ sorbents at high temperature: Experimental and modeling kinetic analysis of the carbonation reaction. J. Environ. Chem. Eng..

[12] Peltzer D., Munera J., Cornaglia L. (2019). The effect of the Li:Na molar ratio on the structural and sorption properties of mixed zirconates for CO_2_ capture at high temperature. J. Environ. Chem. Eng..

[13] Bamiduro F. (2015). Synthesis and Characterisation of Zinc Oxide and Sodium Zirconate particles. Ph.D. Thesis.

[14] Alcanter-Vazquez B., Duan Y., Pfieffer H. (2016). CO Oxidation and Subsequent CO_2_ Chemisorption on Alkaline Zirconates: Li_2_ZrO_3_ and Na_2_ZrO_3_. Ind. Eng. Chem. Res..

[15] Alcerra-Corte L., Fregoso-Isreal E., Pfieffer H. (2008). CO_2_ Absorption on Na_2_ZrO_3_: A Kinetic Analysis of the Chemisorption and Diffusion Processes, *J*. Phys. Chem..

[16] Zhou D., Wang Y., Memon M.Z., Fu W., Wu Z., Sheng S., Zhang H., Ji G. (2022). The Effect of Na_2_ZrO_3_ Synthesis Method on the CO_2_ Sorption Kinetics at High Temperature. Carbon Capture Sci. Technol..

[17] Munro S., Ahlen M., Cheung O., Sanna A. (2020). Tuning Na_2_ZrO_3_ for fast and stable CO_2_ adsorption by solid state synthesis. Chem. Eng. J..

[18] Ampian S.G. (1968). X-Ray and Optical Crystallographic Data for Na_2_ZrO_3_. J. Am. Ceram. Soc..

[19] Bastow T.J., Hobday M.E., Smith M.E., Whitfield H.J. (1994). Structural Characterisation of Na_2_ZrO_3_. Solid State Nucl. Magn. Reson..

[20] Cortes-Palacios L., Collins V.I., Diaz A., Lopez A. (2012). New Mechanism of Sodium Zirconate Formation. Chem. Mater. Res..

[21] Ji G., Memon M.Z., Zhuo H., Zhao M. (2017). Experimental study on CO_2_ capture mechanisms using Na_2_ZrO_3_ sorbents synthesized by soft chemistry method. Chem. Eng. J..

[22] Pavan A.F., Ling C.D. (2022). Phase Formation and Degradation of Na_2_ZrO_3_ under CO_2_ Cycling Studied by Ex Situ and In Situ Diffraction. Inorg. Chem..

[23] Chang R., Menon A.S., Svensson Grape E., Broqvist P., Inge A.K., Cheung O. (2024). Rethinking the existence of hexagonal sodium zirconate CO_2_ sorbent. J. Mater. Chem. A.

[24] Inorganic Crystal Structure Database Physical Sciences Data Science Service. https://www.psds.ac.uk/icsd.

[25] Bos J.-W., Hesse F., Sanna A., Quinn R., Gammie C., Kennedy B. (2019). Determination of the Accurate Crystal Structure of Na2ZrO3 and Its Conversion to Na_2_CO_3_ upon CO_2_ Uptake, STFC ISIS Neutron and Muon Source. https://data.isis.stfc.ac.uk/doi/STUDY/108682279/.

